# Endoscopic transnasal "microsurgery" for suprasellar meningioma involving the anterior communicating artery complex

**DOI:** 10.3171/2025.10.FOCVID25131

**Published:** 2026-01-01

**Authors:** Hirotaka Hasegawa, Shunya Hanakita, Masahiro Shin, Yuki Shinya, Motoyuki Umekawa, Taichi Kin, Nobuhito Saito

**Affiliations:** 1 Department of Neurosurgery, Saitama Medical Center, Saitama Medical University, Kawagoe, Saitama;; 2 Department of Neurosurgery, The University of Tokyo, Bunkyo, Tokyo; and; 3 Department of Neurosurgery, Teikyo University, Itabashi, Tokyo, Japan

**Keywords:** bimanual dissection, endoscopic transnasal surgery, microsurgical technique, planum sphenoidale meningioma, skull base reconstruction

## Abstract

This video demonstrates endoscopic transnasal "microsurgery" for a suprasellar meningioma involving the anterior communicating artery complex. A 44-year-old woman presented with visual loss due to a suprasellar tumor. Preoperative three-dimensional simulation aided in planning the expanded skull base exposure. Using bimanual dissection and meticulous vascular handling, the tumor was resected while preserving critical structures. Skull base reconstruction employed multilayered techniques with dural suturing. This case illustrates how endoscopic microsurgical strategies enable safe resection of anatomically challenging tumors, combining the benefits of endoscopic visualization and microsurgical precision for optimal outcomes.

The video can be found here: https://stream.cadmore.media/r10.3171/2025.10.FOCVID25131

## Transcript

### 0:29 Patient Information.

This video demonstrates endoscopic transnasal "microsurgery" for suprasellar meningioma involving the anterior communicating artery (ACom) complex. The patient is a 44-year-old woman who presented with progressive visual decline in her left eye. Her visual acuity was 20/200 with temporal hemianopsia. MRI revealed a suprasellar mass consistent with a suprasellar meningioma, with significant involvement of the ACom complex. There was tumor invasion into the left optic canal, but not on the right side.

### 0:56 Differences Between Planum Sphenoidale and Tuberculum Sellae Meningiomas.

This slide highlights the anatomical differences between PSM and tuberculum sellae meningioma (TSM).^1^ TSMs typically elevate the optic chiasm and rarely involve the ACom complex, making transnasal access safer and more straightforward. In contrast, PSMs displace the chiasm downward and often involve the ACom complex, requiring vascular dissection and making endonasal surgery more challenging—especially in large tumors. It should be noted, however, that these tumors exist along a spectrum, and in some cases the distinction is not clear-cut; therefore, we refer to our lesion more broadly as a "suprasellar meningioma." My focus today is how to safely perform endoscopic surgery even in these anatomically demanding suprasellar meningioma cases.

### 1:57 Preoperative Three-Dimensional Simulation.

This is a preoperative three-dimensional simulation (GRID; Kompath Inc.) to enhance surgical precision and accuracy. After performing a wide skull base opening, we can appreciate that the optic chiasm is displaced posteroinferiorly, and the ACom complex is involved in the tumor and pushed upward. The red vessel here represents the left Heubner’s artery, running along the posterosuperior surface of the tumor.

### 2:22 Patient Position.

The patient was secured in a supine position with a 15° reverse Trendelenburg tilt to improve venous return. The head was slightly tilted so the nostril faced the surgeon and fixed with a 3-pin skull clamp, with no neck flexion or extension. We used a robotic endoscope holder to enable a stable endoscopic view, allowing true bimanual manipulation. Surgical navigation was preregistered, and the nasal cavity is prepared with topical decongestants.

2:53 Skull Base Exposure. Starting from the left nostril, the middle turbinate (MT) is gently medialized, and the ethmoid air cells are removed to expose the sphenoid sinus. Using the newly created space, the MT and superior turbinate (ST) are then lateralized, thereby fully widening the common nasal meatus while preserving them. The sphenoid ostium (Os) is subsequently widened. The same steps are repeated on the right side. A wide anterior sphenoidotomy is then performed, and the sphenoid mucosa overlying the sella turcica and planum sphenoidale is elevated with its pedicle preserved. The planum sphenoidale, tuberculum sellae (TS), and the superior aspect of the sella turcica, along with both optic canals (OCs), are meticulously exposed using a diamond drill and Kerrison rongeurs.

### 3:44 Tumor Resection.

After opening the dura at the base of the tumor, internal decompression is performed to facilitate tumor mobilization. There is no direct tumor invasion into the right optic canal, as demonstrated on the preoperative MRI. The tumor is meticulously dissected from the surrounding structures such as the right A2 segment and right optic nerve (ON). The left optic canal (OC) dura is opened to access and grasp the anterior margin of the tumor, which is progressively dissected off the ON. The tumor is detached from the diaphragma sellae using bipolar cautery and microscissors. Dissection proceeds by mobilizing the tumor away from the optic chiasm and the lamina terminalis using bimanual technique. Repeated tumor debulking is carried out to enhance subsequent mobilization. Sharp dissection is performed to separate the tumor from the deep vein and the ACom complex. Gentle retraction with the suction tip in the left hand is essential for optimal visualization of dissection planes. The dissection continues along the vascular plane of the left A1 segment, thereby dividing the tumor into two smaller portions to facilitate further removal. During resection of the inferior portion of the tumor, multiple feeding arteries from the left A1 are encountered, which need to be coagulated and sharply divided to prevent avulsion injury. The remainder of the inferior tumor is then completely resected. As dissection continues around the superior portion of the tumor, a feeding branch from the left recurrent artery of Heubner is identified, which is carefully coagulated and divided in a pinpoint manner. Tumor dissection is continued using gentle retraction with the suction tip in the left hand and scissors or a dissector in the right, along with repeated debulking as needed. Meticulous bimanual sharp dissection is essential to avoid injury to perforating vessels. Tumor located posterior to the ACom complex is carefully mobilized and resected, with a small adherent portion deliberately left in place due to tight adhesion. Finally, the optic canal is reinspected to ensure complete tumor removal.

### 6:32 Skull Base Reconstruction.

Reconstruction begins with subdural placement of a collagen sheet (DuraGen; Integra LifeSciences), followed by watertight continuous suturing of an inlay fascia using a 4-0 Kashime suture (Kono Seisakusho Co., Ltd.), initiated at the inferior aspect and advanced in a clockwise fashion.^2^ Upon completion of the suturing, an onlay fascia is placed and covered with sphenoid mucosa harvested earlier during the surgery, then sealed with fibrin glue. To buttress these multilayered reconstruction materials, fat grafts and a sinus balloon are placed.

### 7:12 Postoperative Course.

Postoperative MRI shows gross-total resection of the tumor without evidence of residual enhancement. Despite a tiny asymptomatic high spot in diffusion-weighted imaging, her recovery was uneventful. Importantly, her vision improved significantly after surgery, and no cerebrospinal fluid leakage was observed.

### 7:34 Summary: Surgical Approaches for PSM and TSM.

There are three main approaches to PSM and TSM. Microscopic transcranial surgery offers wide exposure and vascular control but requires brain retraction.^3,4^ Endoscopic keyhole approaches reduce brain manipulation but have limited reach and require advanced skills.^5^ Endoscopic transnasal surgery avoids brain retraction and provides excellent visual outcomes,^3,4,6–8^ but for planum tumors, its use is limited by anterior and lateral extension and frequent vascular involvement. As demonstrated in the video, we introduced the concept of endoscopic microsurgery, which combines endoscopic visualization with microsurgical principles. This involves bimanual dissection within expanded corridors and fine handling of critical neurovascular structures, enabling precise intradural skull base surgery. Bimanual handling is also essential for dural suturing, which significantly improves the durability of skull base reconstruction.^9^ It is notable to say that, while in this case we reconstructed the skull base with multilayered free grafts and watertight dural suturing, a vascularized nasoseptal flap is also a widely accepted and effective option, especially when the risk of CSF leakage is higher. With this approach, we achieve improved lateral reach, enhanced exposure, and better control over vascular structures. Although in some centers a two-surgeon, four-hand technique is employed for efficiency, in this case a robotic holder enabled one-surgeon bimanual dissection, which was also effective.^10^ Finally, regardless of the surgical approach, careful evaluation of optic canal invasion on preoperative imaging and by intraoperative inspection is essential in the management of suprasellar meningiomas, both to ensure oncological completeness and to preserve visual function.

## Disclosures

The authors report no conflict of interest concerning the materials or methods used in this study or the findings specified in this publication.

## Author Contributions

Primary surgeon: Hasegawa. Assistant surgeon: Hanakita, Shin, Umekawa. Editing and drafting the video and abstract: Hasegawa, Kin. Critically revising the work: Hanakita, Shin, Shinya, Umekawa. Reviewed submitted version of the work: Hanakita, Shin, Shinya, Umekawa, Saito. Approved the final version of the work on behalf of all authors: Hasegawa. Supervision: Hanakita, Shin.

## Supplemental Information

### Patient Informed Consent

The necessary patient informed consent was obtained in this study.
